# Integrating Visual and Network Data with Deep Learning for Streaming Video Quality Assessment

**DOI:** 10.3390/s23083998

**Published:** 2023-04-14

**Authors:** George Margetis, Grigorios Tsagkatakis, Stefania Stamou, Constantine Stephanidis

**Affiliations:** 1 Foundation for Research and Technology—Hellas (FORTH), Institute of Computer Science, 70013 Heraklion, Greece; 2 Department of Computer Science, University of Crete, 70013 Heraklion, Greece

**Keywords:** QoE, QoE assessment, video streaming, deep learning, ITU-T P.1203, PatchVQ

## Abstract

Existing video Quality-of-Experience (QoE) metrics rely on the decoded video for the estimation. In this work, we explore how the overall viewer experience, quantified via the QoE score, can be automatically derived using only information available before and during the transmission of videos, on the server side. To validate the merits of the proposed scheme, we consider a dataset of videos encoded and streamed under different conditions and train a novel deep learning architecture for estimating the QoE of the decoded video. The major novelty of our work is the exploitation and demonstration of cutting-edge deep learning techniques in automatically estimating video QoE scores. Our work significantly extends the existing approach for estimating the QoE in video streaming services by combining visual information and network conditions.

## 1. Introduction

According to Cisco’s Visual Network Index report, video accounted for 82 percent of all Internet traffic in 2022, in contrast with 2017, when it occupied 73 percent [1]. Video-on-demand services such as Netflix, YouTube and Amazon Prime, as well as live video services from Twitch, YouTube Gaming, etc., will lead to a global market of 102 billion dollars by 2023 [2]. Several challenges are related to video streaming such as stalls, pixelisation, compression artefacts, changes in rate and rebuffering events, among others [3,4]. The reason for these issues is that video is streamed over different types of networks, both wired and wireless. Resolving these challenges leads to a satisfactory experience which positively impacts customer turnover [5].

Non-streaming video content distribution approaches depended on peer-to-peer networks for progressively downloading videos for later consumption. This is not the case with video streaming, where a large number of subscribers may request video from the server, which leads to bandwidth issues [6]. In addition, due to the fact that viewers pay for having access to video streaming services, they are not tolerant towards the aforementioned issues which affect the quality of the video. This highlights the interest video streaming providers have in assessing the Quality of Experience (QoE) and improving it [7]. According to ITU-T (2017) [8], QoE refers to “the degree of delight or annoyance of the user of an application or service” (p. 25).

The heterogeneity of devices and networks [9] and the need to offer the best possible QoE led to the adoption of Hypertext Transfer Protocol (HTTP) adaptive streaming (HAS) [10], with the most popular adaptive streaming solutions being Dynamic Adaptive Streaming (DASH) over HTTP [11] and HTTP Live Streaming (HLS), by Apple [12]. These consider the Hypertext Transfer Protocol (HTTP) on top of the Transmission Control Protocol (TCP), which constitutes the primary protocol for multimedia content delivery over the Internet [11]. Despite the fact that TCP provides reliable delivery of data leading to the effective transmission of packets, delay of data due to changes to network conditions may exist, thus resulting in affecting video quality [6,13]. Given these conditions, the actions that a provider can take are related to the introduction of Automatic BitRate (ABR) for continually adjusting the quality (i.e., bitrate) of the video. ABR algorithms decide which segment will be played by taking several metrics such as bandwidth, latency and buffer size into account [14].

An important factor for sustaining users’ satisfaction is the continuous and systematic assessment, measurement and quantification of their User Experience (UX) [15], which translates to the quality evaluation of the service/product they are experiencing. Video quality can be assessed by employing video quality assessment (VQA) scores obtained either through subjective or objective methods [16,17]. Regardless of the method employed, researchers have to address the challenge of how to measure the opinion of each user of each video since the topic of video quality is subjective [18]. In subjective methods, humans are involved in measuring the quality of the video [17]. Users are exposed to distorted videos and through this process a mean opinion score (MOS) is derived [19]. Although subjective methods are the most accurate way for VQA [20], they require resources and are time-consuming. This is the reason why objective methods are more attractive to researchers and a lot of work has been done towards developing objective quality metrics [21]. Objective methods can be classified into full reference, reduced reference and no-reference methods [22]. Full-reference methods require the entire video for comparison with the distorted one. In reduced-reference methods, the comparison occurs between the distorted video and part of the original one. In no-reference methods, the original video is not available when assessing the distorted video [22].

A considerable number of studies have focused on VQA methods that have access to the entire original and distorted video and quantify distortions by applying psychophysical characteristics which stem from human visual perception characteristics [23]. In full-reference methods, Peak Signal-to-Noise Ratio (PSNR) [16], Structural Similarity Image Metric (SSIM) [24] and Video Multimedia Assessment Fusion (VMAF), proposed by Netflix [25], are used as the main quality metrics for 2D videos. This class of methods is of paramount importance when adjusting compression parameters. However, they cannot handle the case of no-reference streaming video.

In the case of no-reference VQA, deep learning-based methods are utilized [26]. In general, these methods rely either on hand-crafted features or on automatically extracted features. In video streaming services, what is of essence is a metric that captures the overall satisfaction of users. For the case of streaming, network-related Quality of Service (QoS) metrics such as packet loss, delay and jitter are used to measure the impact of network conditions [27]. However, these metrics cannot be easily translated into quantifying user experience [28]. A significant amount of research has been conducted to understand, measure and model QoE in different video services and in different network environments (e.g., [29,30]). Zhou et al. [31] provide an overview of subjective studies and objective methods for assessing the QoE of adaptive video streaming. They also compare machine learning-based and non-machine learning-based models, proving that the former exhibit better performance. This knowledge can help service and network providers deliver high-quality and cost-effective services while efficiently managing network operations [32].

### 1.1. The ITU-T P.1203 Standard

The need to capture users’ satisfaction of video quality resulted in the development of the ITU-T P.1203 standard, whose purpose is to measure the quality of HAS sessions [33]. The estimation of QoE is achieved by considering aspects such as audiovisual quality, loading delay and stalling [34,35]. Specifically, P.1203 targets HAS-type streaming of segmented H.264-encoded video sessions with lengths between 1 min and 5 min [33,36]. The P.1203 comprises three modules, namely an audio module Pa, a video module PV and an audio–visual integration module Pq [37]. As mentioned in the work of Satti et al. (2017) [37], depending on the available information, the Pv module offers four input classes termed “modes”:

1. Mode 0: Display resolution, frame rate, instantaneous video bitrate
2. Mode 1: All of Mode 0, frame type/frame size (bytes)
3. Mode 2 and 3: All of mode 1. It also involves detailed parsing of partial or complete bitstream.

For video information, P.1203 encodes resolution (in pixels), bitrate (in kbit/s) and frame rate, while the network state is encoded in initial loading delay and stalling events [35]. The ITU-T P.1203 is a bitstream-based model. In bitstream-based methods, the bitstream is analyzed without decoding the video (and/or comparing it to the original one) [38]. While this approach offers the benefit of not requiring a lot of computation time [38], it relies heavily on the specific parameters of each codec and thus faces limitations in scenarios with limited control [39].

### 1.2. Deep Learning in Image/Video QoE Estimation

The disadvantages of subjective and objective methods have led to using machine learning-based methods for QoE prediction [40]. Deep learning is a subcategory of machine learning [41], with convolutional neural networks (CNNs) being one of the most popular and remarkable deep learning networks [42]. CNNs have proved more accurate than other traditional methods and in many cases human annotators in tasks such as image classification and object detection [43]. One benefit of deep learning models is that they can generalize if they are trained on large-scale labeled datasets [44]. However, this constitutes a challenge since there is usually a lack of training data [45].

In several works the capabilities of deep learning in QoE prediction are explored. In [46], Chen et al. (2022) consider the extraction of relevant spatio-temporal features through deep learning for no-reference VQA with the aim to improve the generalization capability of the quality assessment model when the training and testing videos differ in content, resolutions and frame rate. In the work of Zhang et al. (2020) [47], the DeepQoE, an end-to-end framework for video QoE prediction for multiple sources of data, is proposed. The approach considers three steps, namely feature processing, representation learning and QoE prediction, which aim at predicting either discrete (classification) or continuous QoE (regression) scores from multiple inputs including text, video, categorical information and continuous values. In [45], Tao et al. (2019) use a large-scale QoE dataset to study if it can analyze the relationship between network parameters and users’ QoE and the results show that the introduced deep neural network (DNN) approach predicts subjective QoE scores with high accuracy.

Tran, Nguyen and Thang (2020) [48] use the HAS protocol to study QoE estimation for video streaming by taking advantage of a long short-term memory (LSTM) network. The authors propose an open software where they consider five parameters, namely stalling duration, quantisation parameter (QP), bitrate, resolution and frame rate. They evaluate their software against four reference models (Vriendt’s, Yin’s, Singh’s and P.1203) which are outperformed by the proposed solution. The LSTM network architecture for quality prediction in HAS is also proposed in the work of Eswara et al. (2020) [49], where the model they introduce (i.e., LSTM-QoE) shows better performance than other well known models such as ITU-P.1203. In [50], Gadaleta et al. (2017) use a D-DASH framework that employs deep-q learning algorithms. In this work, the authors consider an LSTM cell along with LSTM and their findings indicate that the proposed framework yields better results compared to other adaptation approaches in terms of video quality, stability and rebuffering avoidance.

Finally, several models for deep learning-based no-reference image quality assessment, also known as blind image quality assessment (BIQA), have been proposed, e.g., [51,52,53].

## 2. Materials and Methods

### 2.1. Proposed Framework and Implementation Methodology

In this work, we propose a QoE estimation framework that does not assume that specific QoE monitoring tools are installed by the client. As a result, the entire process of QoE estimation is performed directly on the Content Distribution Server (CDS). The client-side metrics must rely on no-reference QoE estimation approaches when the original video is not available. The objective in our case then is to offer almost real-time tracking of the achieved QoE and suggest appropriate adaptation, i.e., reduction in bitrate. Metrics like average throughput, initial playout delay and buffer level cannot estimate the actual QoE since they do not capture users’ perceptual experience. Furthermore, client-side QoE estimation methods are not appropriate for the real-time aspect of video streaming since the information reported back to the server is always late, i.e., by the time the client executes the QoE estimation and reports back to the server, the network conditions may very well change dramatically. Therefore, the feedback sent back to the server is outdated.

The proposed approach seeks to capture nuanced phenomena of video streaming QoE like the observation that shorter startup delays have little effect on the QoE [49] or that rebuffering events severely influence QoE [3,49]. In addition, viewers prefer lower resolution than interruptions [54]. We explore the “no distorted video” scenario where we do not have access to the decoded video during the QoE estimation process. The proposed solution includes the advantages below:

1. It does not require dedicated services running on the client.
2. It does not introduce additional complexity to the client side.
3. It does not suffer from lag due to the delays caused by the feedback channel.

As a consequence of our design choices, the proposed scheme can work in real-time scenarios (no need for client feedback) and can be employed for prediction under a proactive operating model. Formally, in our model, we consider three entities:

1. The CDS;
2. The network/Internet;
3. The client.

The CDS has access to two sources of input, i.e., visual information encoded in different versions of the same video and up-to-date network parameters. With respect to the visual content, the server has access to different versions which are created by deploying different compression parameters on the H.264 standard. For the compressed stream production, the original videos were compressed using FFmpeg. The considered network parameters include throughput, rebuffering duration and stalling events.

**Figure 1:**
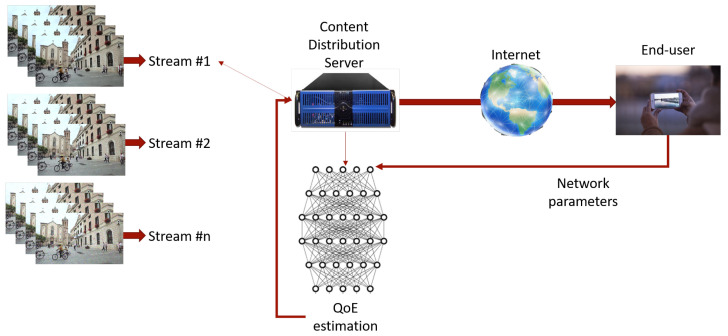
Block diagram of the proposed server-side QoE estimation framework.

The block diagram of the proposed scheme indicating the types of interactions between the client and the server is shown in Figure 1. Effectively, in our scheme, the server has access to different versions of compressed videos and is “as close as possible” to real-time information related to network conditions and, based on these sources of information, it must estimate the user’s QoE.

For the visual features, we consider the PatchVQ [55] model which consists of three stages: (1) extraction of spatio-temporal features; (2) feature pooling; and (3) temporal regression. Spatio-temporal feature extraction occurs by taking into account four scales for each video sequence:

- The entire sequence (full video);
- Spatially localized features (sv-patch);
- Temporally localized features (tv-patch);
- Spatiotemporally localized features (stv-patch).

In all cases, feature extraction is performed by employing DNN-based architectures and more specifically Residual Network (ResNet) and Region-Based CNN (R-CNN) models. The ResNet, which was introduced by Het et al. 2016 [56], applies skip connection and can have a high level of accuracy in feature extraction even in deep networks [57]. The R-CNN is a successful deep leaning technique for object detection because it detects the class of the object and its location [58]. The multilayered hierarchical structures of CNNs allow the extraction of both simple and complex information [59,60]. There are various layers in CNN architectures, but the three main ones for image analysis tasks include convolutional layers, pooling and fully connected layers [61,62]. These types of layers are presented below:

- Convolutional layers: These are responsible for learning the input’s feature representation. These layers consist of several kernels which produce feature maps [61].
- Pooling layers: These layers reduce the height and width of the features and they are applied after the convolutional layers [62].
- Fully connected layers: These layers map the output of the previous layer onto the neuron of the current layer [63].

In this report, regarding spatial features, we consider the features extracted from the PaQ-2-PiQ network [55], a multiscale extension of the 2D ResNet-18 network architecture, which was pre-trained on the LIVE-FB dataset. Furthermore, spatio-temporal features were extracted using a 3D ResNet-18 architecture [64], in which case the model was pre-trained on the Kinetics dataset [65]. Feature pooling was employed in order to reduce the number of trainable parameters and to allow the network to focus on specific regions of interest (ROIs). To extract features, the Faster R-CNN [66] network is considered for both spatial and temporal domains. Faster R-CNN employs a region proposal stage that is considered to select the appropriate regions.

### 2.2. Specifying and Training a DNN Model for QoE Assessment

Deep learning falls under the category of supervized learning. As such, a training dataset needs to be constructed, which plays a significant role in the network’s final performance. The methodology used for effectively optimizing a neural network consists of three main pillars, i.e., a loss function, backpropagation and an optimization algorithm. Using these main ingredients, an iterative process can be constructed to train the network.

After randomly initializing the weights of each layer, the iterative process begins by feeding the training data through the network. This produces an output which is then compared to the expected label with the help of a loss function. This loss function quantifies the error of the network, meaning the degree to which the network can accurately classify the input. The problem of training the network can now be described as the problem of minimizing this error. Given a set of inputs *x*, the DNN produces predicted labels $\hat{y}$ which are evaluated against the ground truth *y* using the $L_{1}$ error metric

(1) $$L\left(x,y\right)=\sum_{i}\left|y_{i}-{\hat{y}}_{i}\right|$$

Given the output of the loss function, a set of error gradients with respect to each of the network’s weights is calculated using the backpropagation algorithm. These gradients are then fed to an optimization algorithm, usually a derivative of gradient descent, which fine-tunes each weight to minimize the error, searching for a local optimum. In this work, we employ the Adam optimizer to train the network.

#### 2.2.1. Proposed Model

**Figure 2:**
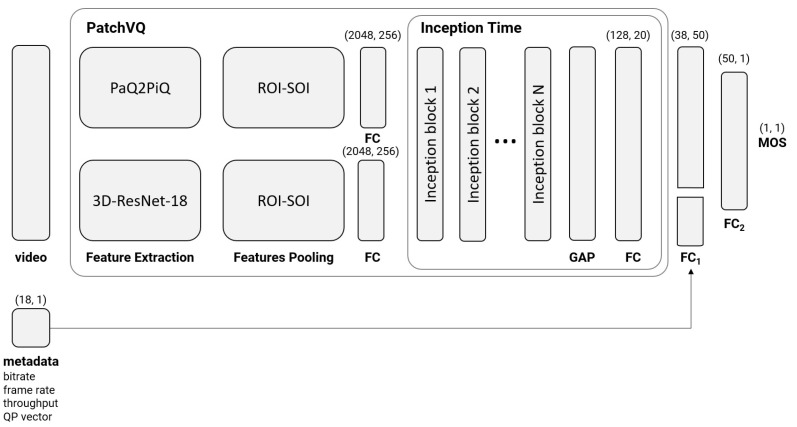
Block diagram of proposed scheme which accepts compressed video segments and associated metadata that produces the MOS.

Our proposed model builds upon the P.1203 [33] and PatchVQ [55] models. In particular, it is a variant of the PatchVQ model. This model involves three sequential steps: feature extraction, spatiotemporal pooling and temporal regression. For spatial feature extraction, the PaQ-2-PiQ [67] backbone is used, while for temporal features a 3D ResNet-18 backbone is used. On the extracted features, a spatiotemporal pooling using a region of interest (ROI) [68] (spatial) followed by a segment of interest (SOI) [69] (temporal) pool approach is applied and the results are then fed to an Inception Time model [70]. PatchVQ has the property of taking into consideration the semantic information of the video features; however, video quality-related metadata such as the encoding QP, bitrate and frame rate, as well as streaming metadata originating from the network over which the video is streamed, such as throughput, can constitute valuable information for inferring the MOS for a given video. To include these features, we had to change the Inception Time component of PatchVQ. The architecture of Inception Time consists of multiple inception blocks, each of which contains multiple parallel convolutions with different filter lengths, followed by a concatenation layer to combine the outputs of the parallel convolutions. The inception blocks are stacked one on top of the other to form the full Inception Time architecture. The output of the Inception Time block network is followed by a Global Average Pooling (GAP) layer and a fully connected layer with a softmax activation function. In our model the final fully connected layer of the Inception Time component was modified to output 20 values instead of one. Then, to those 20 values we concatenated a vector containing: (1) video features, namely the video bitrate, frame rate and a vector of 15 QP values estimated for equally divided segments in the video sequence; and (2) streaming data, i.e., current network throughput. Furthermore, we added another two fully connected layers (FC1, FC2) to enable the model to infer the relations between the MOS and the provided streaming metadata. Our proposed model is depicted in Figure 2.

#### 2.2.2. Model Training

Instead of training the model from scratch, we froze the weights which are provided by Ying et al. in [55] and we only trained the three last fully connected layers. For the training procedure, we employed the $L_{1}$ loss function and the Adam optimization algorithm for a batch size of 128, following the approach in [55].

**Figure 3:**
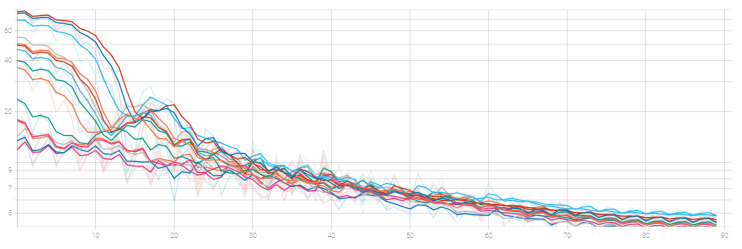
Training loss as a function of training epoch.

For the proposed model training, we used the LIVE-NFLX-II dataset (see Section 2.3), which comprises a total of 420 videos stemming from 15 different uncompressed videos that have been encoded according to the provided dataset information. To train our model, we randomly split the 15 video datasets so that 13 of the original videos are employed for training and two for testing. For each video, all available streamed versions are considered in both training and validation. Figure 3 illustrates the training loss ($L_{1}$ Loss) which indicates its variance across multiple training cycles.

### 2.3. Dataset Analysis

**Figure 4:**
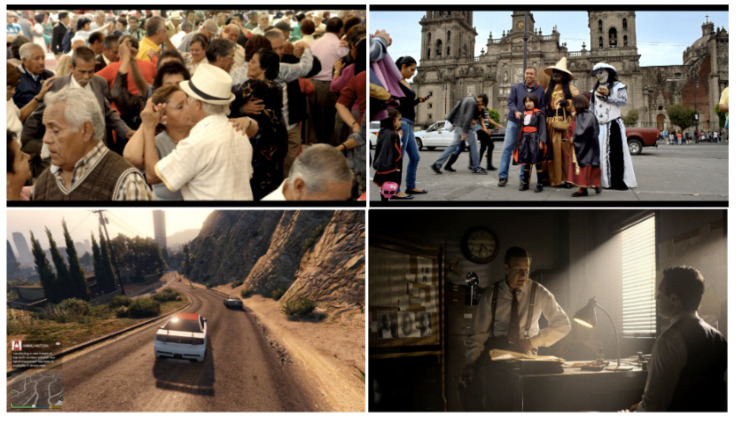
Image samples of the videos used for training.

There are numerous QoE-relevant datasets in the literature. However, very few of them provide the retrospective MOS along with the streaming metadata such as frame rate and bitrate. One dataset that fulfils the prerequisites mentioned above and that was used in this work is the LIVE-NFLX-II dataset [3,71]. Other datasets such as Waterloo Streaming QoE Database III (SQoE-III) [72] do not include continuous QoE scores while the LIVE Netflix Video Quality of Experience Database [73,74], although it provides the retrospective MOS, does not contain network metadata. Typical datasets that have been considered in VQA such as the LIVE Video Quality Challenge (VQC) Database [75] neither contain different sources nor consider different network conditions. LIVE-NFLX-II includes 420 videos that were evaluated by 65 subjects, resulting in 9750 continuous-time and 9750 retrospective subjective opinion scores. Continuous-time scores capture the instantaneous QoE, while retrospective scores reflect the overall viewing experience. These videos were generated from the 15 original videos by considering streaming under 7 different network conditions and employing 4 client adaptation strategies. These 7 network conditions are actual network traces from the High Speed Downlink Packet Access (HSDPA) dataset [76], representing challenging 3G mobile networks. The 4 client adaptation strategies cover the most representative client adaptation algorithms, such as rate-based, buffer-based and quality-based. The selected videos span a wide spectrum of content genres (action, documentary, sports, animation and video games). The content characteristics present a large variety including natural and animation video content, fast/slow motion scenes, light/dark scenes and low and high texture scenes (Figure 4).

The metadata for each video include the following types of information:

1. Four types of no-reference image quality scores (estimated per frame after removing black bars and rebuffered frame), including PSNR, SSIM and VMAF;
2. Information related to the video reproduction such as video and playback duration and number of frames;
3. Information related to visual content including width, height, frame rate, the QP value, scene cuts and the compression bitrate;
4. Information related to network conditions such as rebuffering frames, number of events and duration, throughput and lastly the MOS, both retrospective and continuous.

**Figure 5:**
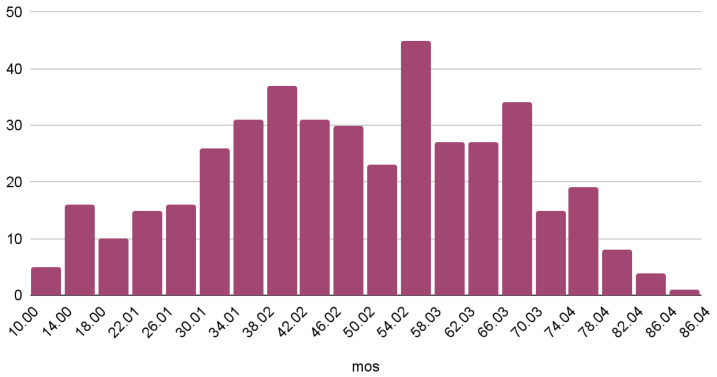
Distribution of the MOS over the entire dataset.

To have a better understanding of the characteristics of the dataset, we performed an analysis relating the MOS with different video and network conditions. Overall, we observed that the MOS is distributed normally with a mean of 48 and a standard deviation of 17; the histogram is presented in Figure 5.

**Figure 6:**
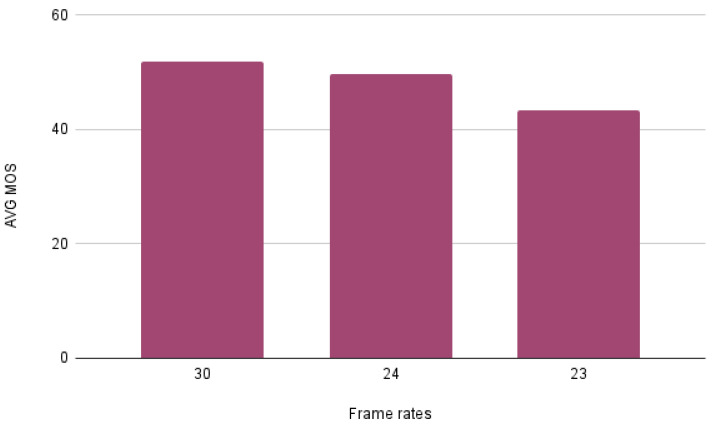
Impact of frame rate of the average MOS.

**Figure 7:**
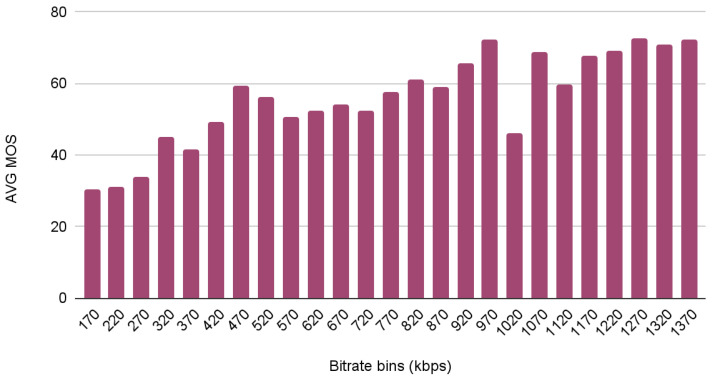
Impact of bitrate on the MOS for video in the dataset.

As can be observed, MOS values follow distribution close to normal distribution, according to which the most frequent subjective opinion score is located in the middle of the scale, while more extreme scores are less likely. Furthermore, we found possible correlations between the MOS and some of the provided metadata. Specifically, we observed that the average MOS has a tendency to increase along with the frame rate and the bitrate, as depicted in Figure 6 and Figure 7 respectively.

It can be easily observed that the highest average MOS is being given to the videos with a frame rate equal to 30 frames per second. Furthermore, it is worth mentioning that the videos’ MOS does not change drastically when the frame rate increases from 24 frames per second to 30.

Figure 7 clearly demonstrates that increasing the bandwidth has a positive impact on quality. Although real observations are inherently noisy, we can observe that the impact is more apparent in the case of very low bitrates, where even small increases have a dramatic effect. On the other hand, we observe that increasing the bitrate above a threshold (around 1Mbit per second) does not appear to affect the MOS to the same extent. This indicates that there are clear quality cut-offs in the low bitrate ranges and there is space for optimization in the case of sufficiently capable network links.

## 3. Results

In order to be able to compare the results yielded by our approach, we proceeded with the evaluation of both P.1203 and the PatchVQ models on the LIVE-NFLX-II dataset. The metrics used for the evaluation are the $L_{1}$ loss, which is a typical loss function used in regression tasks, the linear correlation coefficient (LCC), also known as the Pearson correlation coefficient and the Spearman correlation coefficient (SRCC).

### 3.1. PatchVQ Model

**Figure 8:**
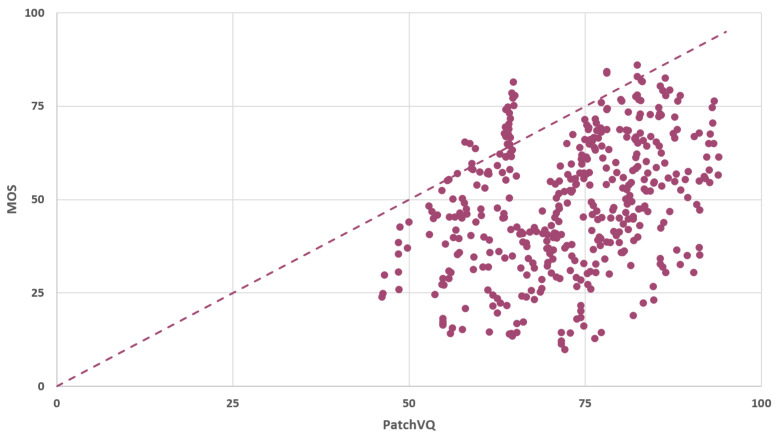
Scatter plot of predicted and measured MOS.

The PatchVQ model implementation is provided by its authors on GitHub. We set up and ran the model according to the guidelines of the authors and we acquired the results in Figure 8.

The rounded mean absolute difference between the predictions and the MOS ($L_{1}$ loss with mean reduction) is calculated to be 23.451 for the investigated dataset. Finally, regarding the histogram of the PatchVQ model’s predictions, a normal distribution of the results was observed with a rounded mean value of 71.119 and a rounded standard deviation of 12.188.

### 3.2. P.1203 Model

The P.1203 standard is provided by ITU. However, there is not an official implementation available. The implementation that we used is available on GitHub and has been verified in terms of performance in relation to subjective test databases created by the authors of the software. Following the guidelines of the authors, we applied the algorithm on the LIVE-NFLX-II dataset for all of the available modes of P.1203.

As the mode increases, the number of inputs that P.1203 takes into consideration increases accordingly. Table 1 presents the inputs of the model for each mode.

**Figure 9:**
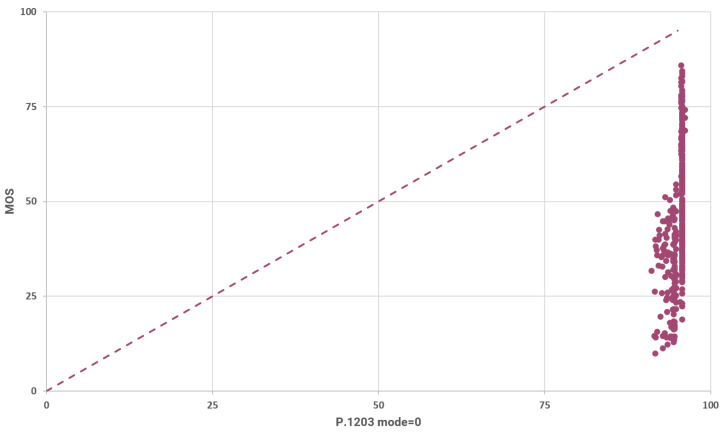
Measured vs. predicted MOS using P.1203 mode 0.

The results that were yielded for all the modes of P.1203 are presented in Figure 9. Figure 9 illustrates the relation between the MOS and the predictions of P.1203 (mode 0). The rounded SRCC for these results is 0.509 and the rounded LCC is 0.546. These metrics indicate a weak positive correlation as can also be inferred from the plot in Figure 9.

**Figure 10:**
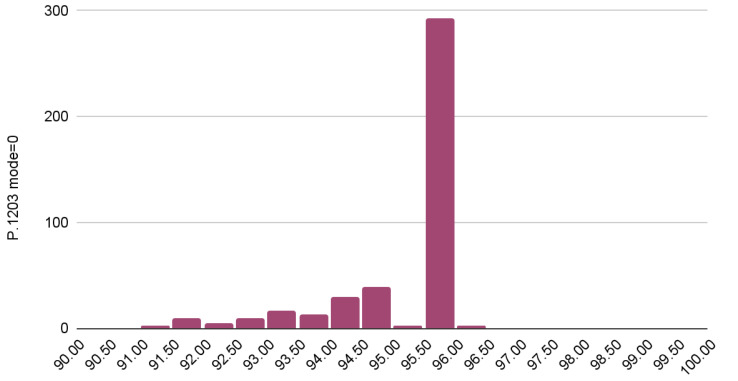
Distribution of estimated QoE using P.1203 mode 0.

The rounded mean absolute difference between the predictions and the MOS ($L_{1}$ loss with mean reduction) is calculated to be 46.242 for these results. The reason that the $L_{1}$ loss is so high can be easily inferred by observing the mean and the variance of the model’s predictions as illustrated in Figure 10. Specifically, the P.1203 model’s predictions (for mode 0) are marginally distributed according to the normal distribution, with a rounded mean value of 95.122 and a rounded standard deviation of 1.015. Clearly, the model predictions in mode 0 are not satisfactory. A reason for this is because the model takes into consideration only the bitrate, the frame rate and the resolution.

**Figure 11:**
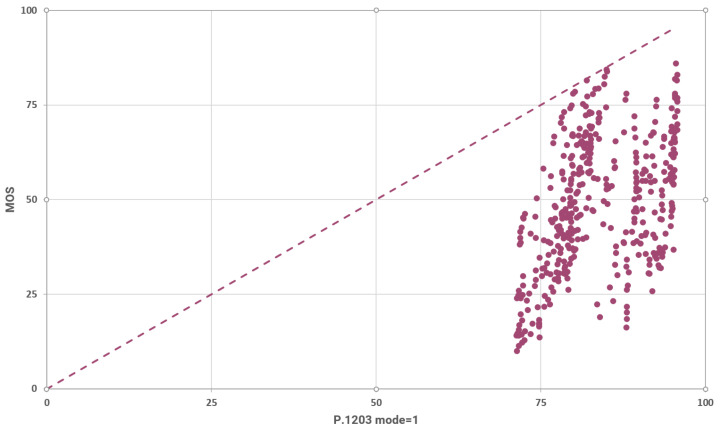
Measured vs. predicted MOS using P.1203 mode 1.

The relation between the MOS and the predictions of P.1203 (mode 1) is visualized in Figure 11. The rounded SRCC for these results is 0.492 and the rounded LCC is 0.459. These metrics indicate a weak positive correlation as well.

The rounded mean absolute difference between the predictions and the MOS ($L_{1}$ loss with mean reduction) is calculated to be 34.974 for these results. The $L_{1}$ loss is decreased by 24% with respect to mode 0, just by adding the extra input features of frame type and frame size. Finally, we calculated the histogram of the P.1203 model’s (for mode 1) predictions and we observed that they are normally distributed with a rounded mean value of 83.855 and a rounded standard deviation of 7.408.

**Figure 12:**
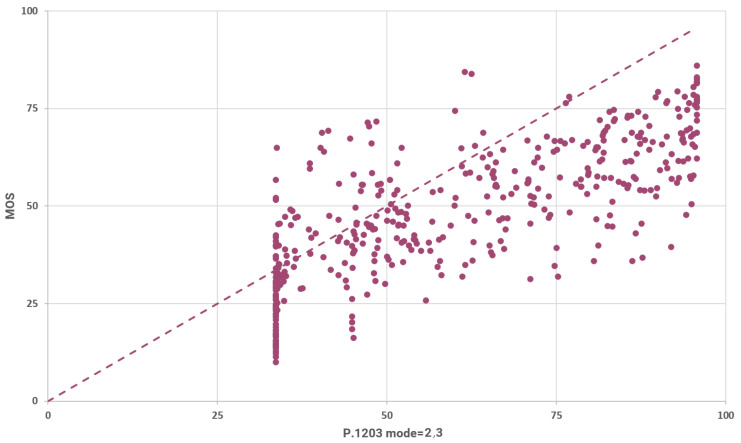
Measured vs. predicted MOS using P.1203 modes 2 and 3.

For the LIVE-NFLX-II dataset the results of the P.1203 in modes 2 and 3 are identical. Thus, we present them both in this section. The relation between the MOS and the predictions of P.1203 (mode 2,3) is illustrated in Figure 12. The rounded SRCC for these results is 0.765 and the rounded LCC is 0.753. These metrics indicate a strong positive correlation as can be inferred from the aforementioned plot.

The rounded mean absolute difference between the predictions and the MOS ($L_{1}$ loss with mean reduction) is calculated to be 15.441 for these results. The $L_{1}$ loss decreases by 56% with respect to mode 1. The P.1203 model’s predictions (for modes 2 and 3) are normally distributed with a rounded mean value of 60.863 and a rounded standard deviation of 21.956. So, in modes 2 and 3 P.1203 achieves its best results.

### 3.3. The Proposed Model

To evaluate our architecture, we performed the evaluation procedure 12 times to make sure that the performance of the model is not a product of variation due to the weight initialization of the model parameters. In Table 2, the metrics for each evaluation cycle are summarized.

The standard deviation and the mean of those metrics are displayed in Table 3 and Table 4, respectively.

**Figure 13:**
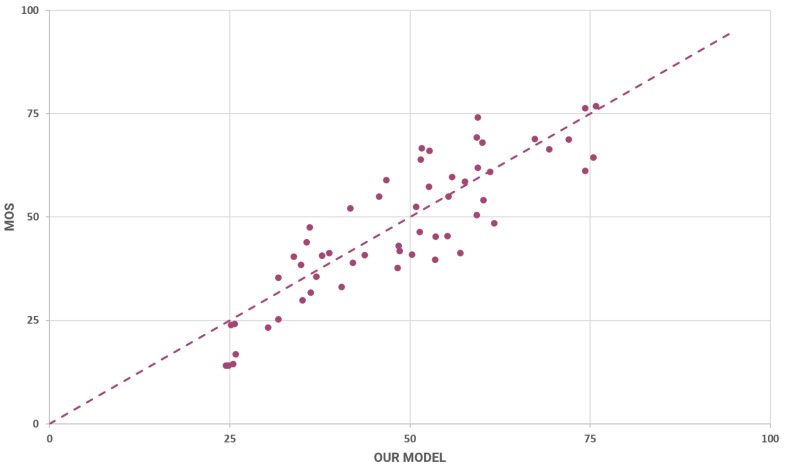
Average predicted vs. measured MOS using the proposed model.

In accordance with the other models, we provide pertinent plots regarding the proposed model’s predictions. Specifically, the results shown in Figure 13 adhere to the evaluation cycle with ID 3 (see Table 2).

The relation between the MOS and the predictions of the model is presented in Figure 13. The rounded mean SRCC for these results is 0.84 and the rounded mean LCC is 0.85. These metrics indicate a strong positive correlation.

### 3.4. Comparison of the Approaches

To compare the aforementioned models, we summarize the metrics for each of them in Table 5.

According to the aforementioned results, the proposed model’s predictions correlate better with the actual MOSs, as is indicated by the greater values of SRCC and LCC. Furthermore, in terms of the mean absolute difference ($L_{1}$ loss), the mean $L_{1}$ loss of the proposed model is considerably less than P.1203’s (modes 2 and 3). In comparison with the PatchVQ model, the proposed model is clearly superior. We attribute this significant increase of the performance to the fusion of both the semantic information of the video and the streaming metadata. We expect that by combining more input features, such as throughput or QP values of the encoder, which are available to the server and in a more efficient manner, the margin between P.1203 and the proposed model could become even wider.

### 3.5. Ablation Study

We ran ablations to explore the effectiveness of our model. We first took network parameters (i.e., throughput) to assess the relationship between the predicted and the measured MOS. We then repeated the process by also taking into consideration visual information, such as QP. The results of the ablation study are provided in Table 6. The ablation studies show that combining visual information and network parameters is necessary for increasing the accuracy of MOS prediction (smaller $L_{1}$ loss).

## 4. Discussion

In this paper, we investigate how deep learning architectures can facilitate the optimization of video streaming by forecasting the user’s experience. The objective, in this case, is to identify the video coding parameters for maximizing the user QoE given visual and network information. The paper analyzes the key issues related to this specific problem and outlines the current landscape in terms of existing and proposed solutions. Given this analysis, we address these issues by introducing multi-modal deep learning architectures. The major novelty of our approach is that the estimation process is executed at the server and thus does not require direct access to the decoded video at the client. To the best of our knowledge, this work is the first machine learning-based method that can simultaneously capture the impact of both video compression and network-related impairment in the user-derived QoE. By leveraging the training dataset, the proposed scheme can act proactively, adapting the streaming characteristics to match the anticipated network conditions, instead of reacting to them.

Given this major difference compared to the state of the art, we propose the exploitation of both visual and network-related information for the automated estimation of the MOS, a reliable proxy to QoE. Overall, the experimental results indicate that the proposed scheme surpasses the performance of both visual-only deep learning methods and network-oriented methods. Specifically, based on the experimental analysis provided in Section 3, we can outline a number of key findings, specifically:

- Approaches that consider network conditions lead to significantly higher prediction performance, compared to visual-only methods when investigating dynamic video streaming conditions.
- Exploiting semantic information encoded in videos through deep learning methods can significantly increase performance, compared to approaches that focus on the networking aspect only.
- It is possible to introduce both visual and network-related information into a unified deep learning model that can be trained in an end-to-end fashion.

Continuing in this line of research, the plans for the following period involve exploring the enhancement of our model by including more parameters that are available on the server side and by introducing contextual, visual and network information. Specifically, motivated by the findings of the proposed approach which indicate that simultaneously encoding visual and network information can lead to higher QoE estimation accuracy, it is interesting to explore how introducing high-level contextual information such as image and video semantics could lead to even higher performance. 

## Figures and Tables

**Table 1:** Inputs for the different modes of ITU P.1203.

Mode 0	Mode 1	Mode 2 ^*^	Mode 3 ^*^
(metadata only): bitrate, frame rate and resolution	(frame header data only): all of mode 0 plus frame types and sizes	(bitstream data, 2%): all of mode 1 plus 2% of the QP values of all frames	(bitstream data, 100 %): all of mode 1 plus QP values of all frames

^*^ The difference between mode 2 and mode 3 is the amount of the QP values extracted from the bitstream. The reason for choosing mode 2 over mode 3 is computational complexity. Since in our case this is not an issue, we group modes 2 and 3 together and assume access to the full bitstream. This way, we consider all available information for each method.

**Table 2:** Metrics per evaluation cycle.

Cycle ID	Test LCC	Test SRCC	Test $L_{1}$ Loss
1	0.90	0.91	5.84
2	0.90	0.89	6.62
3	0.85	0.84	7.47
4	0.94	0.93	4.61
5	0.54	0.55	13.38
6	0.72	0.71	12.65
7	0.75	0.75	9.59
8	0.90	0.90	9.13
9	0.70	0.70	11.0
10	0.78	0.76	10.37
11	0.80	0.77	11.60
12	0.65	0.65	12.10

**Table 3:** Standard deviation.

Test LCC	Test SRCC	Test $L_{1}$ Loss
0.11	0.11	1.75

**Table 4:** Mean values.

Test LCC	Test SRCC	Test $L_{1}$ Loss
0.77	0.76	10.84

**Table 5:** Comparison of the different approaches studied.

	P.1203 m = 0	P.1203 m = 1	P.1203 m = 2,3	PatchVQ	Proposed Model ^*^
LCC	0.55	0.46	0.75	0.42	**0.77**
SRCC	0.51	0.49	**0.76**	0.40	**0.76 **
$L_{1}$ Loss	46.24	34.97	15.44	23.45	**10.84 **

^*^ The metrics of the proposed model are the mean metrics of the 12 evaluation cycles.

**Table 6:** Mean metrics when taking into account network parameters and when combining network parameters and visual information.

	Network Parameters	Network Parameters and Visual Information
LCC	0.77	0.77
SRCC	0.77	0.77
$L_{1}$ Loss	15.88	10.84
